# Transient Triplet Differential (TTD) Method for Background Free Photoacoustic Imaging

**DOI:** 10.1038/s41598-018-27578-9

**Published:** 2018-06-18

**Authors:** Joel W. Y. Tan, Chang H. Lee, Raoul Kopelman, Xueding Wang

**Affiliations:** 1 Department of Biomedical Engineering, University of Michigan, Ann Arbor, Michigan 48109 USA; 2 Department of Chemistry, University of Michigan, Ann Arbor, Michigan 48109 USA; 3 Department of Radiology, University of Michigan Medical School, Ann Arbor, Michigan 48109 USA

## Abstract

With the capability of presenting endogenous tissue contrast or exogenous contrast agents in deep biological samples at high spatial resolution, photoacoustic (PA) imaging has shown significant potential for many preclinical and clinical applications. However, due to strong background signals from various intrinsic chromophores in biological tissue, such as hemoglobin, achieving highly sensitive PA imaging of targeting probes labeled by contrast agents has remained a challenge. In this study, we introduce a novel technique called transient triplet differential (TTD) imaging which allows for substantial reduction of tissue background signals. TTD imaging detects directly the triplet state absorption, which is a special characteristic of phosphorescence capable dyes not normally present among intrinsic chromophores of biological tissue. Thus, these triplet state absorption PA images can facilitate “true” background free molecular imaging. We prepared a known phosphorescent dye probe, methylene blue conjugated polyacrylamide nanoparticles, with peak absorption at 660 nm and peak lowest triplet state absorption at 840 nm. We find, through studies on phantoms and on an *in vivo* tumor model, that TTD imaging can generate a superior contrast-to-noise ratio, compared to other image enhancement techniques, through the removal of noise generated by strongly absorbing intrinsic chromophores, regardless of their identity.

## Introduction

Photoacoustic (PA) imaging is a versatile imaging modality that has been steadily growing in popularity in the last few decades. PA imaging is based on the phenomena of generation of acoustic waves by the absorption of electromagnetic energy^1^. Performed in a label-free manner, PA imaging can describe the morphological structures of biological samples based on the intrinsic optical absorption contrast among various tissues. However, like the use of contrast agents in many other imaging modalities, using optically absorbing contrast agents can make PA imaging more powerful and versatile^2^. While PA imaging enhanced by such contrast agents has progressed significantly since its conception and has shown promise in probing not only structural but also molecular level information in biological samples *in vivo*, one of the main challenges is the presence of various intrinsic body chromophores that produce strong background signals^3,4^. Here, we introduce a new technique called transient triplet differential (TTD) imaging which enables significant reduction of the background signal, regardless of the background chromophores.

**Figure 1:**
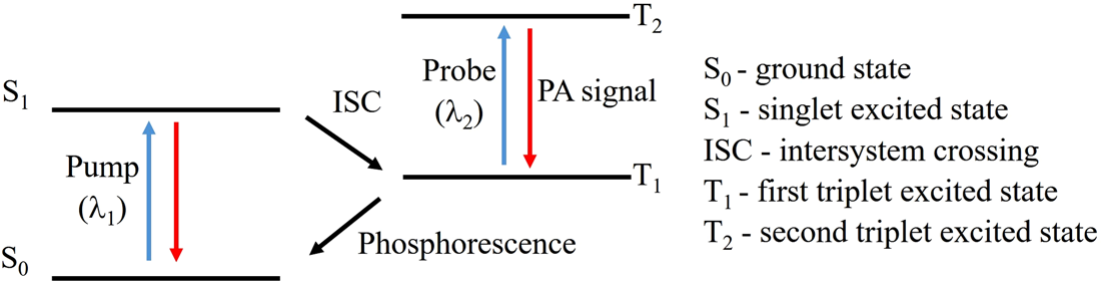
Diagram demonstrating the transition from the ground state to the triplet state. The pump is defined as the laser used to excite the molecules from the ground to the triplet state, while the probe is the laser excitation used to measure the PA signal generated from the transition between the T_2_ and T_1_ states.

Ordinarily, when an organic molecule is excited, it enters an excited singlet state, followed by a decay back into its ground state. This can happen via one of four processes; release of photons (fluorescence), release of heat (vibrational relaxation), product formation (photochemistry), and intersystem crossing (ISC) into the triplet state; the latter decays at a much slower rate, from microseconds to milliseconds, through phosphorescence^5^. In the context of this study, we are focusing on the triplet state because of the presence of the long-lived triplet absorption peak that is only accessible for phosphorescent dyes. The process of generating a TTD signal is demonstrated in Fig. 1, where a “pump” beam is used to excite the molecule from the ground state to the excited singlet state, which will then transition into the triplet state via ISC. Usually, the triplet state has a triplet absorption peak (λ_2_) that is spectrally well-shifted from the original singlet state absorption peak (λ_1_). By using a “probe” beam at this triplet state absorption peak, a PA signal will be generated by molecules that are in the triplet state at that time. Hence, a PA differential signal can be generated by comparing the PA signal at λ_2_ with and without a “pump” beam. Since only triplet state molecules of the phosphorescent dye will generate this PA signal, the differential signal will purely originate from the phosphorescent dye. This will allow for the removal of all background signals from any biological or other relevant chromophores, as these are non-phosphorescent. We define such a PA differential signal as the TTD signal. The technique introduced in this manuscript is similar to transient absorption spectroscopy (TAS), which uses both “pump” and “probe” beams, except that TAS is often concerned with the excited singlet state rather than the triplet state^6^.

Besides giving phosphorescence, molecules in the triplet state can also return to the ground state via quenching by other triplet state molecules, such as oxygen, which exist naturally in the triplet state^7^. This process further limits the lifetime of the phosphorescent dyes in the triplet state, where it has been shown that the triplet state molecules will decay near-exponentially in time for phosphorescent molecules such as methylene blue (MB)^8–10^. The decay curves are affected by the oxygen concentration and this is an area of active research, where studies, including some focusing on PA methods, are being conducted to classify and characterize the change in the decay curves with respect to the oxygen concentration, with the end goal of mapping oxygenation levels in tissue^7–14^.

In this study, we used methylene blue conjugated polyacrylamide nanoparticles (MBNP) by using the advantages from this nano-platform that has been developed extensively^15,16^. MB is known to have a high quantum yield for ISC into the triplet state^17,18^. MB can exist in two states, a monomer and a dimer state, with different ISC efficiencies. The dimer state has a triplet excited state lifetime (10^−8^ s) that is much shorter than that of the monomer state (10^−6^–10^−4^ s)^17^. The relative monomer and dimer state populations are dependent on the concentration of the MB in the media and it is very challenging to approximate correctly the optimal concentration of free MB molecules *in vivo* (maximal monomer signal with minimal dimer signal). On the other hand, the monomer/dimer ratio in MBNP is not affected by the concentrations of the nanoparticles (NPs) because the MB to NP matrix ratio is always constant^19^. As shown in Fig. S1, the monomer/dimer ratio in MBNP was measured to be approximately 1:1. A constant monomer/dimer ratio facilitated by the polyacrylamide NPs makes the lifetime-based functional measurement quite robust, even in a complex *in vivo* environment. In addition, MB is known to degrade into colorless “leuko-methylene blue” through enzyme reductions occurring in the bloodstream; while the NP matrix can effectively protect the MB from degradation in the presence of those enzymes^15^. The nanoparticle matrix can be surface modified with a targeting moiety, such as an F3-peptide or other peptides^19–22^. This allows the delivery of the NPs to specific tissues, or cells, *in vivo*^19,21,23,24^. Besides that, certain sizes of NPs (50–100 nm), and specifically of these hydrogel NPs, are also known to accumulate well in tumors, due to the enhanced permeability and retention (EPR) effect^25^. All these advantages associated with the use of the polyacrylamide nano-platform could be highly valuable for future translation of the proposed technique to preclinical or clinical settings.

While triplet state imaging has been used as a method for measuring oxygen levels^7–14^, its application was not focused on producing background free PA imaging. We compare both a single wavelength contrast-agent enhanced image, and one of the more current PA background reduction techniques, called *spectral unmixing* or *multi-wavelength imaging*^26–28^, with our newly developed TTD imaging, for both phantom and an *in vivo* study. Our results suggest that the TTD imaging technique can produce background free PA images even in the presence of strongly competing chromophore signals, which has not been achieved by the other two contrast enhancement techniques.

## Results

### TTD signal for MBNP

**Figure 2:**
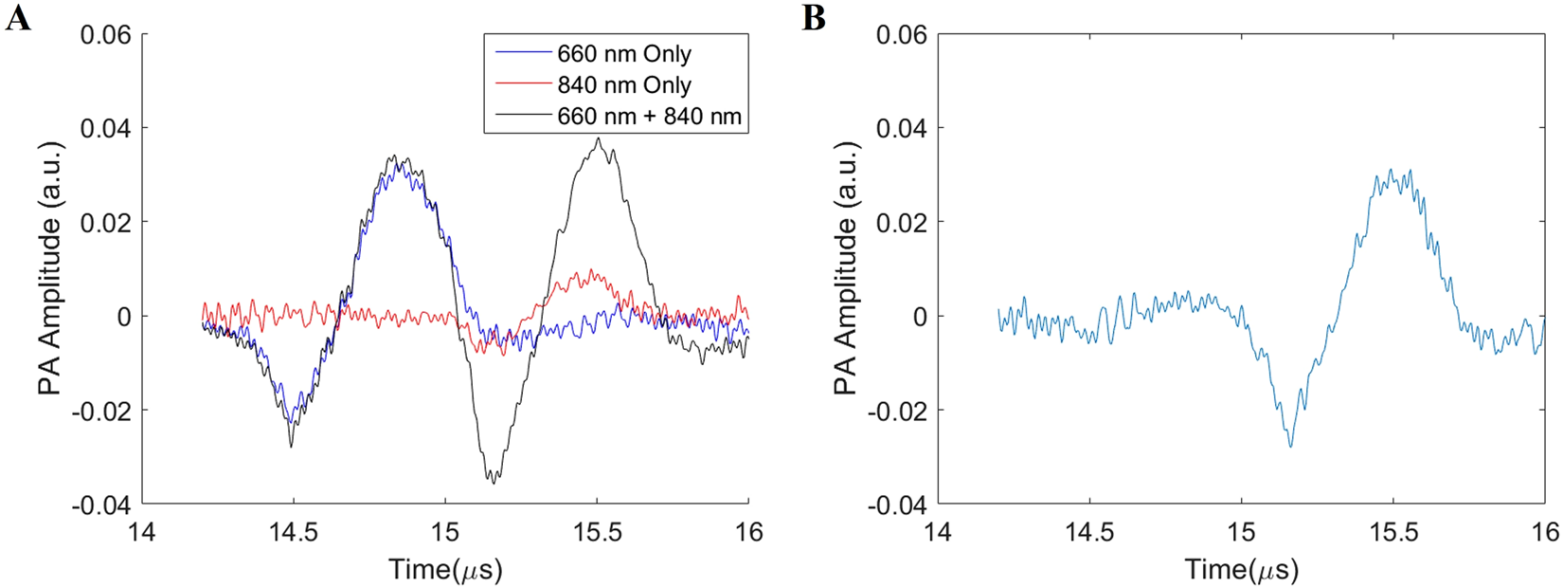
(**A**) Superimposed PA signals from the individual wavelength measurements for the pump (660 nm), probe (840 nm), and from the pump plus probe (660 nm + 840 nm) wavelengths. (**B**) TTD signal obtained by subtracting the individual 660 nm and 840 nm signals from the 660 nm + 840 nm signal for a 2.0 mg/mL MBNP sample in a visible-light transparent tube with a 0.5 μs delay time.

Figure 2 demonstrates the process of obtaining the TTD signal. Figure 2A shows the superimposed waveforms (PA signals) obtained for the pump (660 nm), probe (840 nm), and pump plus probe (660 nm + 840 nm) excitations. Here, there is a noticeable increase in the signal amplitude for the second peak in the pump plus probe waveform, which corresponds to the waveform shown in Fig. 2B. This *increase in signal* is the TTD signal. The used wavelengths of 660 nm and 840 nm are the peak singlet and triplet state absorption wavelengths, respectively, for the MBNP, which is shown in Fig. S2. Since the delay time used is on the order of 1 μs, the TTD signal is solely due to MB in the monomeric state as all the MB in the dimer state would have returned to the ground state due to their much shorter triplet state lifetime (10^−8^ s). Previous works have identified the monomeric MB absorption peak at 660 nm and the triplet absorption peak at 840 nm^17^, which matches with what was experimentally found in Fig. S2.

### Factors affecting TTD imaging

**Figure 3:**
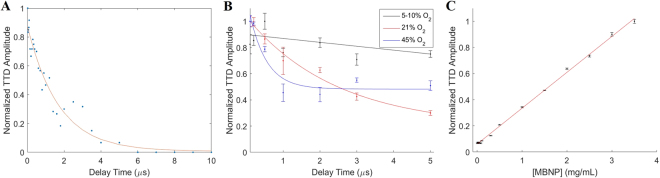
Normalized TD signal for (**A**) Varying delay times, with exponential decay curve fit of r^2^ = 0.9485, (**B**) Low (5–10%), air (21%), and medium (45%) oxygen levels of the MBNP sample solution, and (**C**) Varying [MBNP] in PBS. Error bars indicate the standard deviation of the signal for 5 repeated measurements.

**Figure 4:**
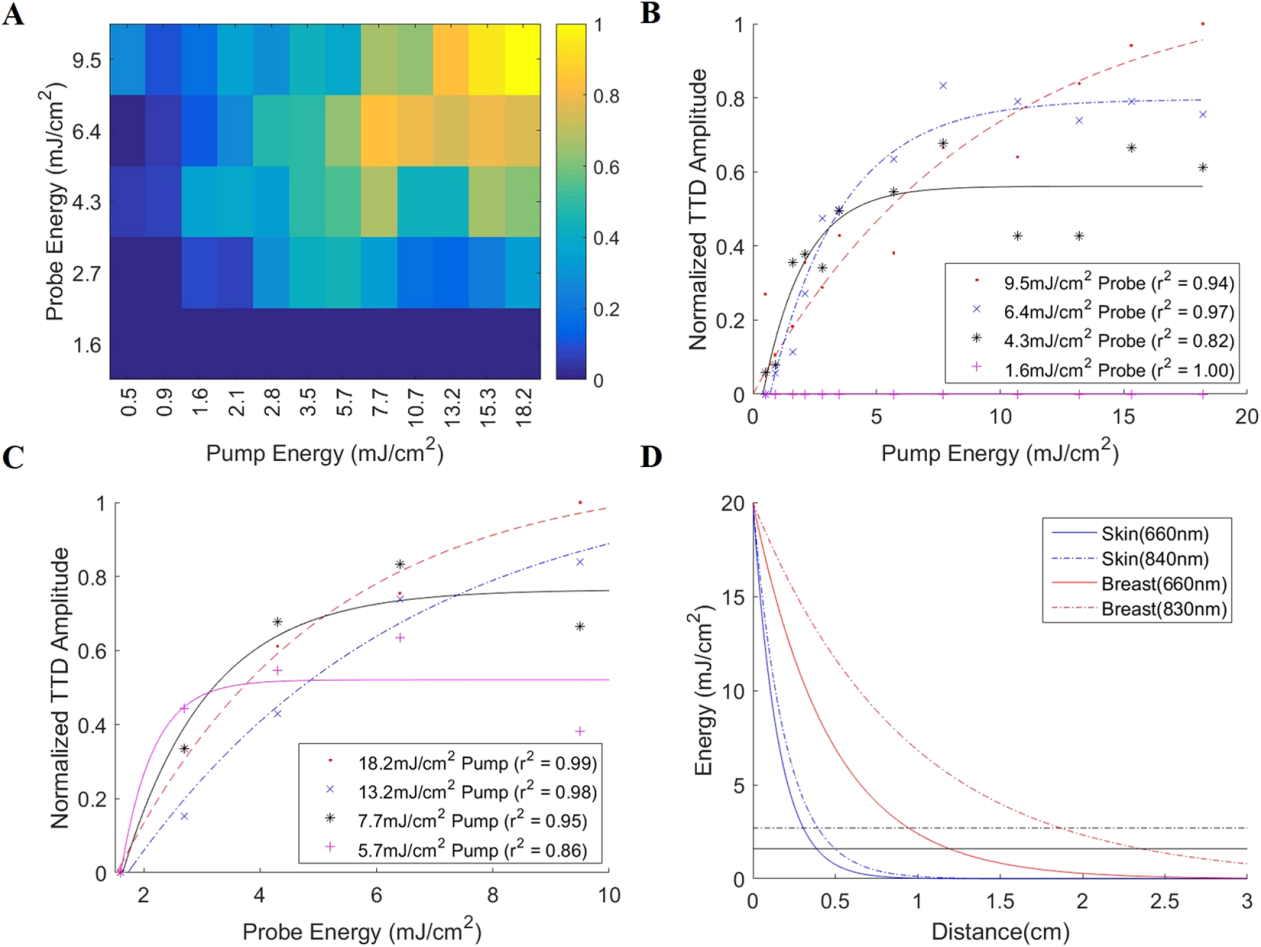
Energy dependence of the TTD amplitude, with (**A**) showing the normalized TTD amplitude 2-D plot vs pump and probe energy, **(B**,**C)** showing the normalized TTD amplitude vs pump/probe energy at 4 fixed probe/pump energy levels respectively, and (**D**) showing the simulated energy vs penetration depth for skin and breast tissue with initial fluence of 20 mJ/cm^2^ (maximum permissible exposure limit). Colorbar in (**A**) shows the normalized intensity based on the maximum TTD intensity. The black lines (solid – 660 nm, dashed – 840 nm) in (**D**) indicate the minimum energy required for TTD imaging for the respective wavelengths, derived from (**A**) where the minimum pump energy is taken as 1.6 mJ/cm^2^ and the minimum probe energy as 2.7 mJ/cm^2^.

We looked at the effects of multiple parameters on the TTD signal of MBNP, specifically the delay time between the pump and probe lasers, oxygen level, MBNP concentration, and the pump and probe laser energy. Two other factors that also affect the TTD signal, that of the laser wavelengths and the monomeric-dimer ratio of the MBNP, are not discussed in Figs 3 and 4 as the former has already been determined for monomeric MB, while the latter is fixed in the case of the MBNP.

Figure 3A demonstrates the relationship between the TTD amplitude and the delay time for MBNP from 0.02 μs to 10 μs. The TTD amplitude follows a decaying exponential relationship with increasing delay time as reported in previous studies^9,11^. From Fig. 3B, we see that the slope of the exponential decay of the TTD amplitude increases as the oxygen concentration increases. As mentioned previously, since oxygen exists naturally in the triplet state, the higher the concentration of oxygen, the more triplet oxygen molecules are available to quench the triplet state MB molecules. This leads to a faster decay in the TTD amplitude as the concentration of oxygen increases. At low oxygen levels (5–10%), we see an almost linear relationship between the TTD amplitude and the delay time, which has been previously reported^13^. Figure 3C shows that the TTD amplitude has a linear relationship with the concentration of MBNP, as expected.

Theoretically, we expect the TTD amplitude to vary linearly with pump and probe energy. The greater the pump energy, the greater the amount of MB molecules that are pumped into the triplet state, and hence the greater the TTD amplitude. Similarly, the greater the probe energy, the greater the amount of triplet state molecules that are excited, and hence the greater the TTD amplitude. This linear relationship only holds until saturation, since the maximum TTD amplitude is dependent on both pump and probe energies, and one or the other will act as a limiting factor. In the case of excess probe energy, the limitation factor becomes the number of triplet state molecules that are available to be excited, which is determined by the pump energy. In the case of excess pump energy, the limitation factor becomes the energy available to excite the triplet state molecules, which is determined by the probe energy.

Figure 4A shows the general trend: As the pump and probe energy increases, the TTD amplitude increases. Figure 4B shows the relationship between the TTD amplitude and probe energy for various fixed pump energies. For low probe energies, the TTD amplitude increases approximately linearly with pump power before reaching a plateau. As the probe energy increases, the pump energy level at which the TTD amplitude plateaus increases. At the highest probe energy used, the TTD amplitude never reaches a plateau within the experimental range of pump energy values used. This means that within the experimental values used, the MBNP is never fully saturated. Looking at the two plateau values for 4.3 mJ/cm^2^ and 6.4 mJ/cm^2^ probe energies, the TTD amplitude decreases by 29% for a 33% decrease in probe energy, which supports the linear relationship between the TTD amplitude and the probe energy. Figure 4C shows a similar relationship between the TTD amplitude and probe energy for various fixed pump energies. Looking at the two plateau values for 5.7 mJ/cm^2^ and 7.7 mJ/cm^2^ pump energies, the TTD amplitude decreases by 32% for a 26% decrease in pump energy. Figure 4D shows the simulated penetration depth for TTD imaging in the skin and breast using available optical absorption and scattering information from literature^29–32^. These two tissue types are shown to demonstrate the feasibility of using TTD imaging in, for example, skin and breast cancer related diseases. Here, the estimated maximum penetration distance for TTD imaging (based on the maximum permission exposure limit of 20 mJ/cm^2^) is approximately 3.9 mm for the skin and 12 mm for breast tissue, where, in both cases, the pump wavelength (660 nm) acts as the limiting energy factor, due to the fact that 660 nm light cannot penetrate as well as 840 nm light in optically scattering biological tissues.

### Contrast-to-noise ratio and sensitivity

**Figure 5:**
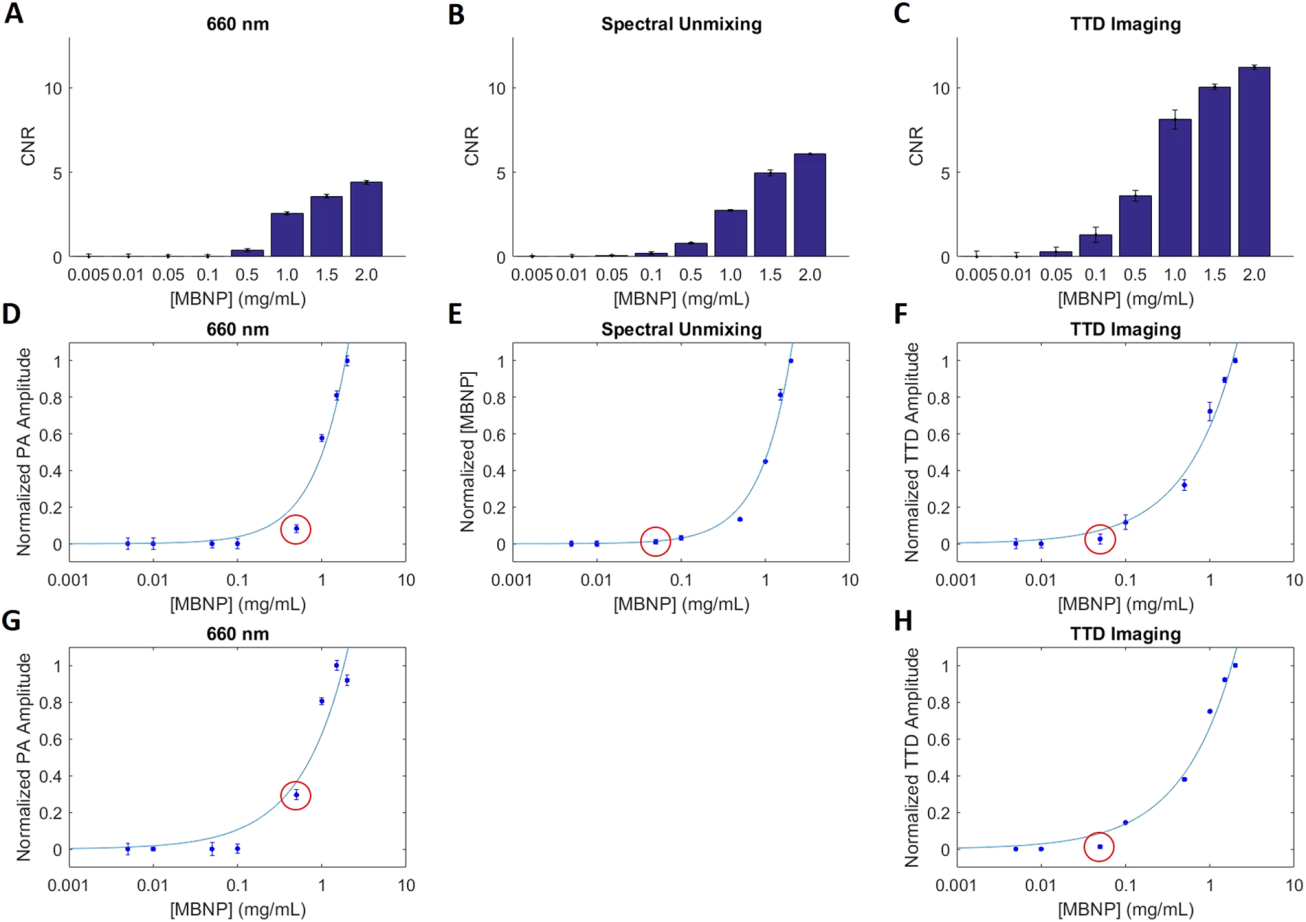
CNR and sensitivity measurements comparing 3 methods; MBNP, as a contrast agent, taken at its absorption peak of 660 nm (**A**,**D**,**G**), spectral unmixing (SU) (**B**,**E**), and TTD imaging (**C**,**F**,**H**). The CNR in (**A–C**) where measured by taking the difference between the PA signal at a given concentration and the PA signal at zero concentration of MBNP, divided by the maximum noise fluctuation. Figures **(D**–**H)** show the minimum detectable concentration of MBNP (sensitivity) which are labeled by the red circles. Figures (**A**–**F**) corresponds to MBNP suspended in 5% blood to mimic physiological conditions, while (**G**,**H**) are suspended in water. 5% blood was used as most biological tissues contain between 0–5% of blood by volume^32^. Spectral unmixing was not conducted for the MBNP in water due to the absence of deoxyhemoglobin and oxyhemoglobin in the sample. Error bars indicate the standard deviation of the signal for 5 repeated measurements.

In terms of contrast-to-noise ratio (CNR), Fig. 5A–C show that TTD imaging clearly has the superior CNR at all detectable concentration levels, followed by spectral unmixing (SU), and subsequently by the MBNP contrast agent at 660 nm. The CNR for TTD imaging is approximately double that of SU at all concentrations, and triple that of the MBNP contrast agent alone. Figure 5D–F show the sensitivity of the various imaging techniques for MBNP in the presence of 5% blood, while Figs 5G,H show the sensitivity of MBNP in the presence of water. Here, we found that TTD imaging has approximately the same sensitivity levels in *both* blood and water, being able to detect up to 0.05 mg/mL of MBNP. This high sensitivity level is significant as it allows for its potential use *in vivo*, where this concentration would involve a 400 times dilution of a typical injection volume using 20 mg/mL MBNP. This means that even if the target area receives a concentration of only 1/400 of the injected MBNP, the sensitivity offered by TTD imaging will still be high enough to detect it. In addition, TTD imaging demonstrates a 10x superior detection sensitivity compared to that of just using the MBNP contrast agent, and shows detection sensitivity similar to that of SU. The TTD amplitude itself is also unaffected by the blood, as can be seen from having similar PA amplitudes for the various concentrations in blood and in water (Fig. 5F,H). While TTD imaging seems to have a similar sensitivity to SU, the body has many more additional chromophores beyond the two chromophores accounted for of deoxyhemoglobin (Hb) and oxyhemoglobin (HbO_2_), such as melanin, lipids, etc., which will make SU significantly more challenging *in vivo*.

### Effectiveness in different background noises

**Figure 6:**
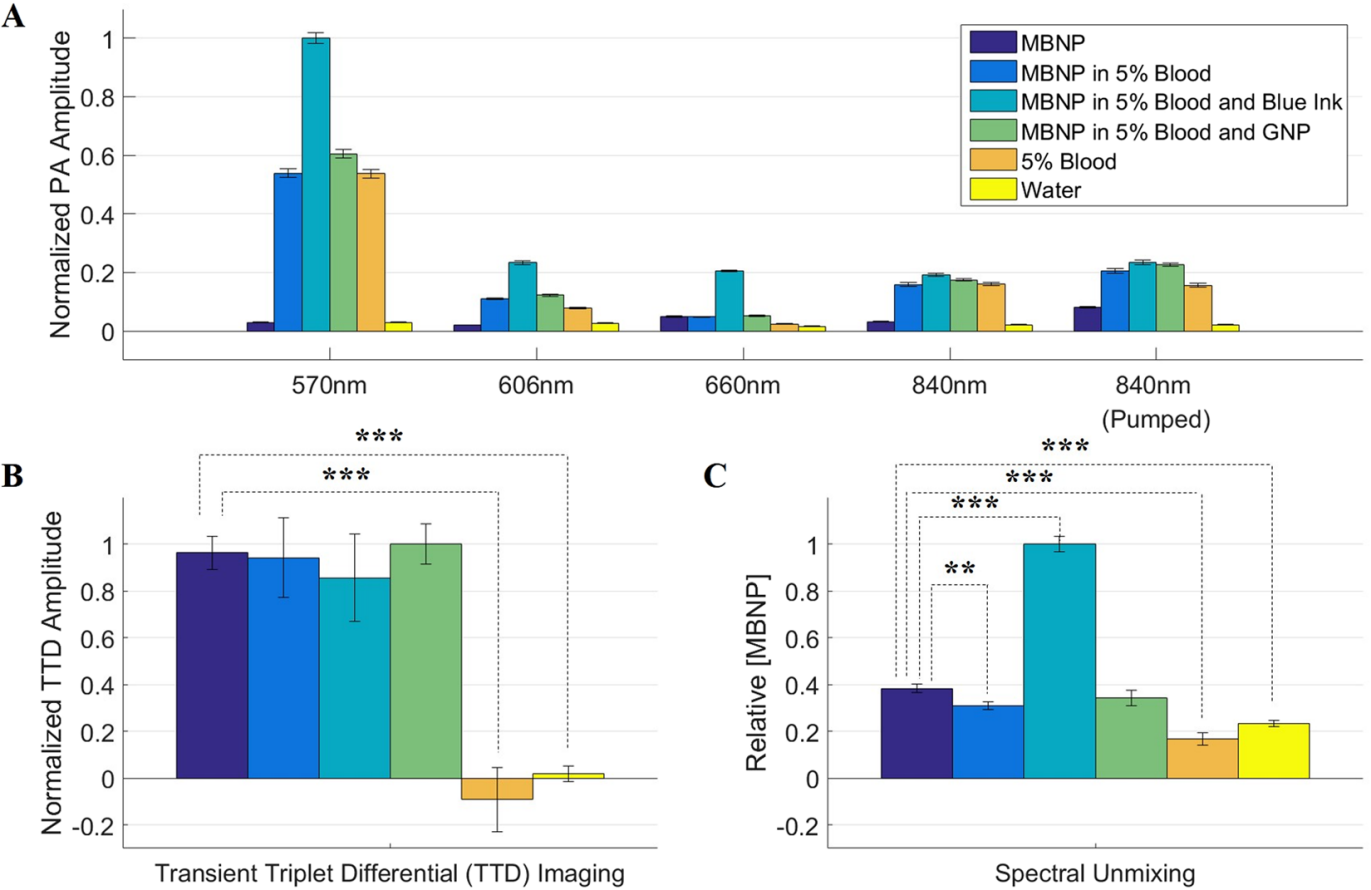
Data comparing the TTD imaging method and SU for six different samples, including 2.0 mg/ml MBNP, 2.0 mg/ml MBNP in 5% whole blood, 2.0 mg/ml MBNP in 5% whole blood plus 0.1% blue ink, 2.0 mg/ml MBNP in 5% whole blood and 5% gold nanoparticles (GNP), 5% whole blood, and water. Both the 5% blood and the water samples act as the controls. (**A**) Normalized PA amplitude at 5 separate wavelengths. (**B**) Normalized TTD amplitude. (**C**) Relative MBNP concentration measured by spectral unmixing. Error bars indicate the standard deviation of the signal for 5 repeated measurements. **represents p < 0.01, and ***represents p < 0.001 for a t-test with hypothesis that the normalized TTD amplitude/relative [MBNP] of the sample is equal to that of the MBNP only sample.

Figure 6A shows the PA amplitudes of the six different samples at the 5 chosen wavelengths reflecting the spectroscopic absorption property of each sample. As expected, samples with 2.0 mg/ml MBNP show a higher absorption at 660 nm as compared to the controls. The blue ink (0.1%) acts as an unknown chromophore which significantly increases the absorption at 570 nm, 606 nm, and 660 nm and acts as an increased background noise at those wavelengths. Samples containing 5% whole blood show a higher absorption at all the wavelengths except at 660 nm which is dominated by the MBNP signal. The sample containing 5% gold nanoparticles (GNP) with a peak absorption wavelength at 532 nm acts as the second unknown chromophore, showing a small increase in PA amplitude at the 570 nm and the 606 nm wavelengths due to residual optical absorbance. Figure 6B shows that there is no statistical difference between the TTD amplitude obtained for the four samples containing MBNP, which suggests that the TTD amplitude is independent of the background chromophores present in the sample. Importantly, the two control samples did not show any TTD signal, which demonstrates the potential of TTD imaging for removing background noise. This is contrasted with the SU method in Fig. 6C, which shows a baseline signal even in the absence of any MBNP. Furthermore, the SU method shows a significantly increased error in the estimated MBNP concentration for the sample containing the blue dye, as it was indistinguishable by the SU algorithm from the MBNP, since the blue dye and the MBNP have similar absorption profiles for the chosen wavelengths, as shown in Fig. S1. The SU method also shows a significant difference for the 2.0 mg/mL MBNP in 5% blood sample, which is probably due to errors in the unmixing algorithm, arising from ignoring all other chromophores beyond the MBNP, Hb, and HbO_2_. These unaccounted-for chromophores include water as well as the transparent PVC tubing used to hold the sample. This result on phantoms demonstrates the ability of TTD imaging to isolate the MBNP signal from those of both known and unknown background chromophores. The presence of these chromophores is almost inevitable for any *in vivo* imaging application. A similar example in the presence of a muscle tissue background is shown in Fig. S4.

### Validation on an *in vivo* mouse tumor model

**Figure 7:**
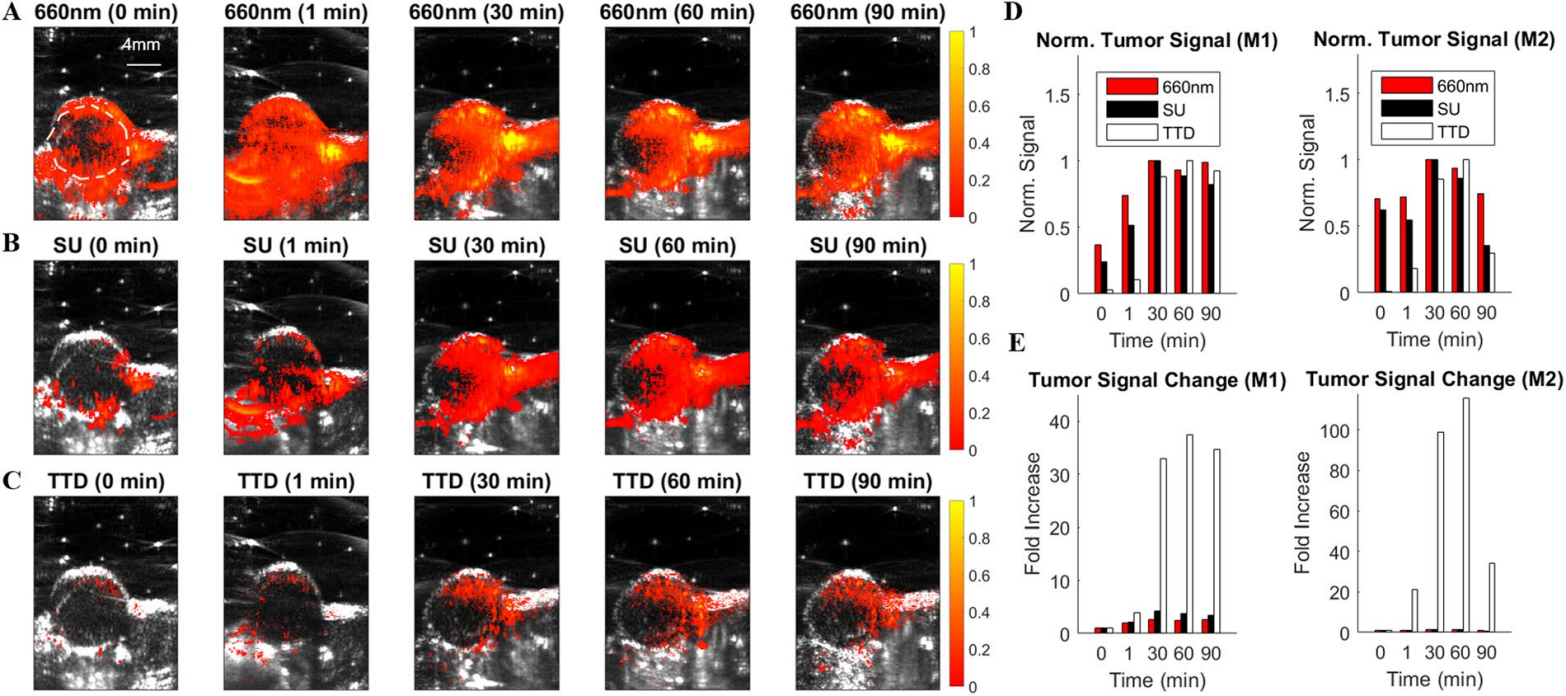
*In vivo* time-lapse images of a mouse subcutaneous tumor model using: (**A**) a single wavelength at 660 nm, (**B**) MBNP concentration as determined via SU, and (**C**) TTD imaging. Each PA image is superimposed on the B-scan ultrasound image in gray scale. Time scales indicate the time after MBNP injection, with 0 min being just before the injection. The ROI shown at time 0 min in (**A**) was chosen to encapsulate the whole tumor except the tumor boundaries, which typically generate a much larger PA signal compared to the rest of the tumor. The colorbar shows the normalized intensity based on the maximum intensity within each imaging method. (**D**) Normalized mean tumor signal as a function of time within the ROI indicated in (**A**). (**E**) Mean tumor signal change based on the mean tumor signal at time 0 min. M1 and M2 indicates two separate animal experiments that were conducted. The figures shown in panels **(A**–**C)** are from M1.

To demonstrate the applicability of the TTD technique *in vivo*, we show time-lapse images of a subcutaneous mouse tumor model. F3-conjugated MBNPs were injected intraveneously and preferentially accumulate in the tumor through both F3-targeting^23,24^ and the EPR effect. Figure 7A–C show the superimposed, time-lapsed ultrasound and PA images of the tumor at a single wavelength of 660 nm (Fig. 7A), MBNP concentration as determined via SU (Fig. 7B), and TTD imaging (Fig. 7C). The image at time 0 shows the tumor before the MBNP injection, while the image at time 1 shows the tumor immediately after injection, while 30, 60, 90 mins designate the times after the injection, respectively. Here, we see that at time 0, the TTD image shows an almost zero signal, with increasing signals as time progresses, due to accumulation of the MBNP in the tumor. In contrast, we see some baseline signal at time 0 for both the 660 nm and SU image, although it is significantly smaller in the case of the SU image, and a similar increase in signal as time progresses. There is peak accumulation of the MBNP in the tumor at around 30–90 mins, where the signals in all 3 imaging methods are relatively consistent. It should be noted that most of the TTD signal is localized within the tumor, while there are background signals for the other two methods. However, the majority of the TTD signal is located towards the upper region of the tumor, which is most likely an indicator of the energy constraints of this technique. Figure 7D shows the normalized mean tumor signal based on the region-of-interest (ROI) shown in Fig. 7A. Figure 7E shows the change in the mean tumor signal with respect to the mean tumor signal at time 0 min. Figure 7D,E together show the superiority of the TTD imaging method in removing background signals compared to the other two methods, as demonstrated by the low signal in the tumor at times 0 and 1 min, and the corresponding order of magnitude increase in tumor signal after MBNP accumulation.

## Discussion

While TTD imaging is dependent on many different parameters, most of the parameters can be controlled or accounted for so as to ensure a consistent TTD signal. The pump and probe laser wavelengths, delay times, and monomer/dimer ratio of the MBNP can be controlled and optimized so as to maximize the TTD signal. The first two are modifiable parameters of the lasers while the monomer/dimer ratio can be controlled by modifying the composition of the MBNPs. The oxygen dependence of the TTD signal can be reduced by using a short delay time, during which the TTD signal can be assumed to be approximately invariant to oxygen, as demonstrated in Fig. 3. The energy dependence of the TTD signal is harder to control, due to effects such as light scattering and absorption. To minimize energy effects on the TTD signal, an energy level that saturates the TTD contrast agent should be used, if possible. If a TTD contrast agent can be designed such that it has a lower saturation energy, it could potentially minimize the energy dependence of the TTD signal. In addition, since the TTD signal follows a linear relationship with concentration, the amplitude of the TTD signal should be a direct indicator of the concentration of the MBNP, with deviations only resulting from differences in energy and oxygen levels. Despite the TTD imaging being limited by its energy requirements, the effective imaging depths offered by the current design of the MBNP are estimated as 3.9 mm for skin and 12 mm for breast tissue. In addition, we have demonstrated that we can obtain the TTD signal for an imaging depth of at least 5 mm in an *in vivo* mouse model.

It should be noted that the microsecond scale of the delay time between the pump and the probe light pulses can potentially lead to an overlap of the PA signals from the two pulses, since acoustic waves travel at a much slower velocity than light waves. This could potentially cause problems such as a reduction in the dynamic range of the system due to the increase in the maximum amplitude of the combined signal. In addition, interference between the pump and the probe PA signals could occur. A potential solution to this would be to design a TTD contrast agent with a longer delay time such that the two PA signals do no overlap. An example of a TTD contrast agent such as this would be the oxyphor G2 dye which has a lifetime of up to 250 μs^11^. It should be noted, however, that while this might solve the above-mentioned issues, it could potentially lead to other problems. A longer lifetime also means a longer image acquisition time which could be a problem for time variant systems, for example when the body motion of the imaging subject cannot be ignored. The above-mentioned dynamic range issue can also be solved by calculating the TTD signal in an analog circuit before conversion into a digital system, so as to maximize the dynamic range of the system in detecting the TTD signal.

While we have used MBNP for this proof-of-concept study, there are in fact many other organic dyes capable of phosphorescence that are available for use^5^. Organic dyes, while only forming a subset of PA contrast agents, hold a better chance for clinical translation due to their better biocompatibility and potentially less toxicity. In fact, we chose to use MB for this study as it is both an FDA-approved dye, and a very commonly used contrast agent. That being said, there has been research into triplet states for inorganic dyes, such as gold nanoclusters, although their triplet state absorption was not studied extensively^33^. This TTD technique is unique in the sense that it enhances contrast agents that are already widely used in PA imaging to realize molecular level studies of cancer and a variety of other diseases (with MB itself being a common PA contrast agent). In particular, we expect this method to be widely applicable and to have a broad impact in the cancer imaging field, especially for the targeted imaging of superficial or quasi-superficial cancers, such as skin, head and neck, gastrointestinal track, and urethral track cancers. In this study, we have demonstrated the feasibility of achieving this, using our tumor-targeting F3-peptide conjugated MBNP for subcutaneous tumor imaging. However, since this TTD technique relies on triplet state absorption, there could be potential damage to the cells if the concentration of the phosphorescent dye and the laser energy is high enough. This happens because there is interaction between the triplet state dye and the triplet state oxygen, leading to the formation of reactive oxygen species. Because the main application that we are envisioning for this method is in targeted imaging of cancer, this potential biological damage to the surrounding cancer cells during TTD imaging could actually be beneficial. In other words, the TTD-based PA imaging powered by MBNP could be a theranostic platform for both diagnosis and treatment of cancer.

In summary, the experimental results on MBNP demonstrate the ability of TTD imaging to provide background free PA images in a way that was not achieved by the other two previously established image enhancement methods. TTD imaging has a 3× superior CNR and 10× higher sensitivity than the method using a single-wavelength contrast agent enhanced image. Furthermore, TTD is independent of the background chromophores, while SU is significantly affected by background chromophores with optical absorption spectra similar to those of the chromophore of interest. Unlike SU, TTD imaging does not require knowledge of the spectra of the background chromophores in order to remove them from the image signal. In addition, there will be a non-zero baseline signal for SU, if additional wavelength measurements are not performed to account for all the chromophores present in the sample. While not shown in the results, in theory, the TTD method will allow for the use of multiple contrast agents without having to worry about spectral overlapping. As long as one of the parameters (pump wavelength, probe wavelength, or lifetime) are different, the signals from each respective dye can be isolated. Lastly, SU also takes a significantly longer post-processing time due to the calculations needed to be performed, and can also potentially take a longer acquisition time, depending on the number of wavelength measurements needed to be performed. While SU is an established processing tool for most optical imaging techniques, TTD imaging could prove more useful in complicated biological systems with many different competing chromophores, where the above ability to remove the contributions of all these chromophores and isolate the TTD signal from the phosphorescence dye can be highly valuable in applications such as in molecular imaging of cancerous tissue, in which we have demonstrated the feasibility of accomplishing using our F3-conjugated MBNP in an *in vivo* subcutaneous tumor mouse model.

## Methods

### MBNP preparation

All chemicals were purchased from Sigma-Aldrich unless otherwise noted. The MBNPs were prepared as in a previously reported method^18^. All reactions were performed in the dark. The monomer solution was prepared as follows. DCMB-SE (European Molecular Precision Biotech, 5 mg dissolved in 100 μL of DMSO) was added into 0.93 mL of Phosphate Buffered Saline (PBS, pH 7.4) containing acrylamide (368 mg) and N-(3-aminopropyl)-methacrylamide hydrochloride (Polysciences, 28 mg). The monomer solution was stirred for 2 hours at room temperature. Then, sodium dioctylsulfosuccinate (1.07 g) and Brij 30 (2.2 mL) were added into 30 mL of Hexane in a round bottom flask equipped with a stirring bar. After 30 min of argon flushing, the monomer solution was injected and flushed with argon for another 15 min. The radical polymerization was initiated by addition of 100 μL of N,N,N′,N′-tetramethylethylenediamine and 100 μL of ammonium persulfate (15 mg/100 μL in water), while stirring. After 2 hours, the hexane was evaporated with a rotary evaporator and the resulting MBNP NPs were suspended in Ethanol and transferred into an Amicon Stirred Ultrafiltration Cell equipped with a Biomax 300 kDa membrane. The solution was washed with ethanol and water several times to remove any unreacted monomers and surfactants. Then, the MBNP NPs water suspension was freeze-dried and stored at −20 °C.

For surface modifications, the MBNP solution (50 mg/2.5 mL of PBS pH 7.4) was mixed with 4 mg of bifunctional Polyethylene glycol (MAL-PEG-SCM, 2 kDa, Creative PEGWorks). After 30 minutes of stirring, it was washed with PBS (4 times) using Amicon Ultra Centrifugal Filter (100 kDa) and 11 mg of F3 Peptide (KDEPQRRSARLSAKPAPPKPEPKPKKAPAKKC, RS Synthesis) was added and stirred overnight. Then, cysteine (0.63 mg) was added and stirred for 2 hours to deactivate unreacted maleimide groups. The MBNP solution was washed with water and lyophilized. The MBNP was characterized by UV-VIS spectrometer (Shimadzu), fluorescence spectrometer (Horiba FluoroMax-3), and dynamic light scattering (Beckman Coulter).

### General PA imaging setup

**Figure 8:**
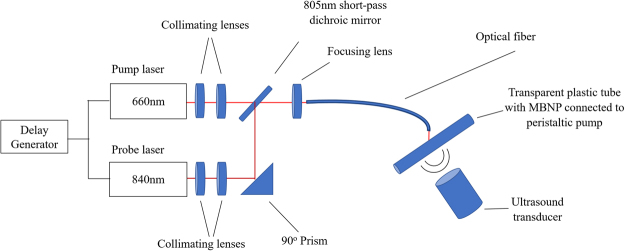
General experimental setup for PA imaging experiments.

In all the experiments, the TTD signal was obtained by taking 3 separate PA signal measurements, specifically with a pump only laser, probe only laser, and a pump plus probe laser separated by a delay. The TTD signal is then obtained by subtracting the pump only and probe only measurements from the pump plus probe measurement. The general experimental setup for all the *in vitro* experiments is shown in Fig. 8.

### Factors affecting TTD Signal

#### Delay time

Using the setup shown in Fig. 8, the TTD signal was measured for various delay times. The pump laser used was a tunable pulsed laser (Surelite OPO Plus, Continuum) pumped by the third harmonic of an Nd:YAG laser (Surelite, Continuum), with pulse duration of 5 ns, and tunable wavelengths in the range of 410–680 nm and 710–2500 nm. The probe laser was produced by an optical parametric oscillator (Vibrant B, Opotek, Carlsbad, CA, USA) pumped by the second harmonic output of a Nd:YAG pulsed laser (Brilliant B, Quantel, Bozeman, MT, USA), with pulse duration of 5.84 ns, and tunable wavelengths in the range of 680–950 nm and 1200–2400 nm. The transparent PVC tube was filled with 3.9 mg/mL MBNP and circulated using a peristaltic pump to ensure a continuous fresh supply of dye. An air pump was connected to the MBNP reservoir to maintain a constant level of oxygen. A section of the tubing was immersed in water to enable acoustic coupling with the ultrasound (US) transducer. A V312 cylindrically focused US transducer (Panametrics), with a central bandwidth of 10 MHz and focal length of 0.75 inch was used to detect the PA signal from the MBNP. The transducer was connected to an oscilloscope (TDS 540, Tektronix Inc) with a sampling rate of 250 MSa/s. The pump and probe wavelengths were fixed at 660 nm and 840 nm, respectively, while the delay time between the pump and the probe laser pulses was set to 0.02, 0.04, 0.06, 0.08, 0.10, 0.15, 0.20, 0.25, 0.30, 0.35, 0.40, 0.50, 0.60, 0.70, 0.80, 0.90, 1.00, 1.20, 1.40, 1.60, 1.80, 2.00, 2.50, 3.00, 3.50, 4.00, 5.00, 6.00, 7.00, 8.00, 9.00, and 10.00 μs using a delay generator (DG535, Stanford Research). Each measurement was recorded five times, and the mean of the recordings were taken to minimize the noise due to the random fluctuations in the signal. The data was processed using Matlab.

#### Oxygen

To alter the oxygen concentrations, an additional tube connected to a gas (oxygen or nitrogen) tank was added to the peristaltic pump reservoir. Additionally, an oxygen meter (Microx TX3, Presens) was immersed into the reservoir to continually monitor the oxygen levels during the experiment. Three different oxygen levels were maintained, specifically at low oxygen (5–10%), air oxygen (21%), and medium oxygen (45%) concentrations. The low oxygen level was maintained by continually adding nitrogen gas into the reservoir, which resulted in an oxygen level of approximately 5–10%. The air oxygen level was maintained by continuously pumping air into the reservoir using the air pump. The medium oxygen level was maintained by adding both air using the air pump and oxygen using the oxygen tank and balancing the gas pressures to form a stable oxygen level. The TTD signals for 7 delay times of 0.1, 0.2, 0.5, 1.0, 2.0, 3.0, and 5.0 μs were measured at each oxygen level for a MBNP concentration of 2.0 mg/ml.

#### MBNP concentration

The delay time was fixed at 0.2 μs. The MBNP concentration was varied between 0.005 mg/ml and 3.5 mg/ml.

#### Pump and probe laser energy

The concentration of MBNP was diluted to 0.6125 mg/ml in order to attempt saturation of the TTD signal within the maximum permissible exposure (MPE) limit. The energy of the pump laser was varied in the range of 0.5–18.2 mJ/cm^2^ on the sample surface while the energy of the probe laser was varied in the range of 1.6–9.5 mJ/cm^2^. The simulated energy vs penetration depth at each wavelength for each tissue was calculated using the effective attenuation coefficient, μ_eff_, and Beer’s law, as shown in equations (1 and 2).

1 $${\mu }_{eff}=\sqrt{3{\mu }_{a}({\mu }_{a}+{\mu }_{s^{\prime} })}$$

2 $$\varphi (z)=\varphi (0){e}^{-{\mu }_{eff}z}$$

where μ_a_ is the absorption coefficient (cm^−1^), μ_s_′ is the reduced scattering coefficient (cm^−1^), and ϕ(z) is the energy at depth z. The values for μ_a_ and μ_s_′ were obtained from previously published results^29–32^.

### CNR and sensitivity

To examine CNR and sensitivity, the concentration was varied in the range of 0.001–2.0 mg/ml for the MBNP with the same conditions as before. Two sets of concentration measurements were conducted, one with the MBNP suspended in distilled water, and the other with the MBNP suspended in 5% human blood to act as biological noise. The PA signals were compared between the three different methods, including 1) the single-wavelength (660 nm) PA amplitude increase due to the increasing MBNP concentration, 2) the spectrally unmixed signal, and 3) the TTD signal. The 660 nm PA amplitude increase was measured by subtracting the PA signal for a given MBNP concentration by the PA signal for a control sample where the concentration of MBNP is zero. The spectrally unmixed signal was calculated by measuring the PA signals at the three wavelengths of 570 nm, 606 nm, and 660 nm, respectively. In this case, the spectrally unmixed signal only considered Hb, HbO_2_, and MBNP. From the available absorption spectra of Hb and HbO_2_, the relative concentration of the MBNP in the PA image can be calculated using the simultaneous equations (3–5).

3 $$k([Hb]{a}_{Hb@570nm}+[Hb{O}_{2}]{a}_{Hb{O}_{2}@570nm}+[MBNP]{a}_{MBNP@570nm})=P{A}_{570nm}$$

4 $$k([Hb]{a}_{Hb@606nm}+[Hb{O}_{2}]{a}_{Hb{O}_{2}@606nm}+[MBNP]{a}_{MBNP@606nm})=P{A}_{606nm}$$

5 $$k([Hb]{a}_{Hb@660nm}+[Hb{O}_{2}]{a}_{Hb{O}_{2}@660nm}+[MBNP]{a}_{MBNP@660nm})=P{A}_{660nm}$$

where k is a constant depending on the Grüneisen parameter of the tissue, the light fluence, and the sensitivity of the imaging system, [x] is the concentration of chromophore x, a_x@λ_ is the absorption coefficient of chromophore x at wavelength λ, PA_λ_ is the photoacoustic signal at wavelength λ.

A constraint where the estimated concentrations had to hold positive values was imposed on the spectral unmixing algorithm. These equations also assumed a constant fluence level at the different laser wavelengths.

The TTD signal is obtained using the method specified in the beginning of the methods section. The CNR was measured by taking the PA signal for the given MBNP concentration and subtracting it from the PA signal at zero MBNP concentration, and dividing it by the standard deviation of the five measurements made for each concentration. The sensitivity was classified as the MBNP concentration before which the PA signal fell below the noise level.

### Effectiveness in different background noises

To study the effects of background noise, various samples were prepared, specifically, 2.0 mg/ml MBNP, 2.0 mg/ml MBNP in 5% blood, 2.0 mg/ml MBNP in 5% blood and 0.1% blue ink, 2.0 mg/ml MBNP in 5% blood and 5% GNP, 5% blood, and distilled water. The GNP was obtained from IMRA America, Inc. The percentages listed are all measured by volume. The samples were prepared by taking a stock solution of 25 mg/mL MBNP and diluting it in PBS together with the other solutions to get the required concentrations. The PA measurements at the five separate wavelengths, 570 nm, 606 nm, 660 nm, 840 nm, and 660 nm + 840 nm, were obtained. The TTD and SU signal were obtained in the same way as previously mentioned.

### Validation on an *in vivo* mouse tumor model

All procedures on live animals were performed in accordance with institutional guidelines and approved by the Institutional Animal Care and Use Committee (IACUC) at the University of Michigan. 9 L glioma cells (ATCC) were cultured in RPMI 1640 media and supplemented with 10% fetal bovine serum and 1% antibiotic-antimycotic. 5-week old athymic nude fox/NU (Envigo) were subcutaneously injected with approximately 10^6^ glioma cells (ATCC) in a 100 μL suspension of culture media. The tumor was allowed to grow for 2–3 weeks until it reached approximately 1 cm in diameter. For imaging, a setup similar to Fig. 8 was used, where instead of coupling the beam to an optical fiber, a freely propagating beam was used. A commercially available research ultrasound platform (V1, Verasonics, Kirkland, WA) using a high frequency linear array transducer with central frequency of 11.25 MHz (CL15-7, Philips, Andover, MA) was used to acquire the ultrasound and PA images from the mice. A delay time of 0.5 μs between the pump and the probe lasers was used. The mouse was placed under anesthesia using 1–2% isoflurane mixed with oxygen for the duration of the experiment and intravenously injected with 0.3 mL of 20 mg/mL of F3-MBNP. The PA measurements at the five separate wavelengths, i.e. 570 nm, 606 nm, 660 nm, 840 nm, and at 660 nm + 840 nm, were obtained for time points 0 (before the injection), 1 (right after the injection), 30, 60, and 90 minutes. To improve the SNR of the images, the images were averaged 50 times. The TTD and SU signals were obtained in the same way as previously mentioned.

### Data availability

The data that support the findings of this study are available from the corresponding author upon reasonable request.

## Electronic supplementary material

Supplementary Figures
