# Supplementary material for: Transient Triplet Differential (TTD) Method for Background Free Photoacoustic Imaging
Source: Sci Rep. 2018 Jun 18;8:9290. doi: 10.1038/s41598-018-27578-9 (PMC6006254; doi:10.1038/s41598-018-27578-9)
Supplement: Supplementary file 1 — Supplementary Figures [file 41598_2018_27578_MOESM1_ESM.docx]

**Transient Triplet Differential (TTD) Method for Background Free Photoacoustic Imaging**

**Joel W.Y. Tan^1^, Chang H. Lee^2^, Raoul Kopelman^1,2,*^, and Xueding Wang^1,3,*^**

^1^Department of Biomedical Engineering, University of Michigan, Ann Arbor, Michigan, 48109, USA.

^2^Department of Chemistry, University of Michigan, Ann Arbor, Michigan, 48109, USA.

^3^Department of Radiology, University of Michigan Medical School, Ann Arbor, Michigan, 48109 USA.

^*^R.K. and X.W. are the corresponding authors. Email: R.K. ([kopelman@umich.edu](mailto:kopelman@umich.edu)); X.W. ([xdwang@umich.edu](mailto:xdwang@umich.edu)).

**Supplementary Figures**


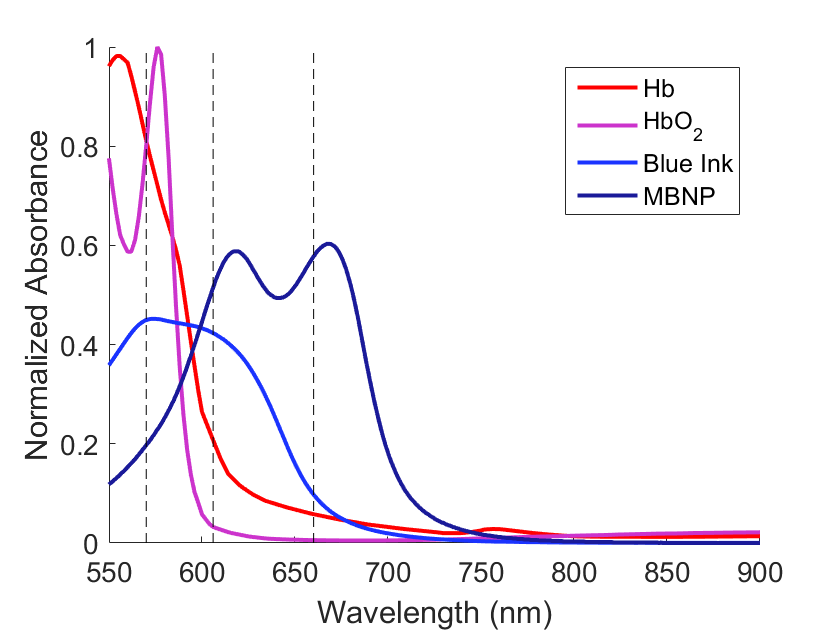


**Figure S1.** Absorption spectra of deoxyhemoglobin (Hb), oxyhemoglobin (HbO_2_)_,_ MBNP, and the blue ink as measured by a UV-VIS spectrophotometer. The approximately 660 nm peak corresponds to the monomer state while the 620 nm peak corresponds to the dimer state, with both states having an approximately equal absorption. The dashed black lines correspond to the three wavelengths used for spectral unmixing. The blue ink and the MBNP have a similar absorption profile in the sense that they show the same trend as compared to Hb and HbO_2_, that is they both have a higher absorption at 606 nm and 660 nm and a lower absorption at 570 nm than Hb and HbO_2_.


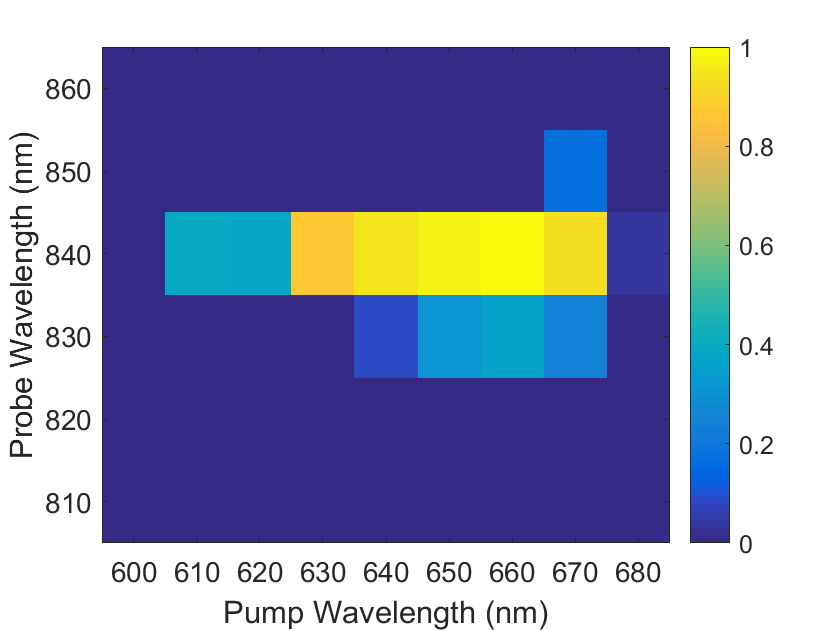


**Figure S2.** Wavelength dependence of the MBNP as measured by PA imaging. Colorbar indicates the normalized TTD signal. The peak TTD signal occurs at 660 nm (pump) and 840 nm (probe) which are the same as the result previously reported in the literature for monomeric MB ^18^.


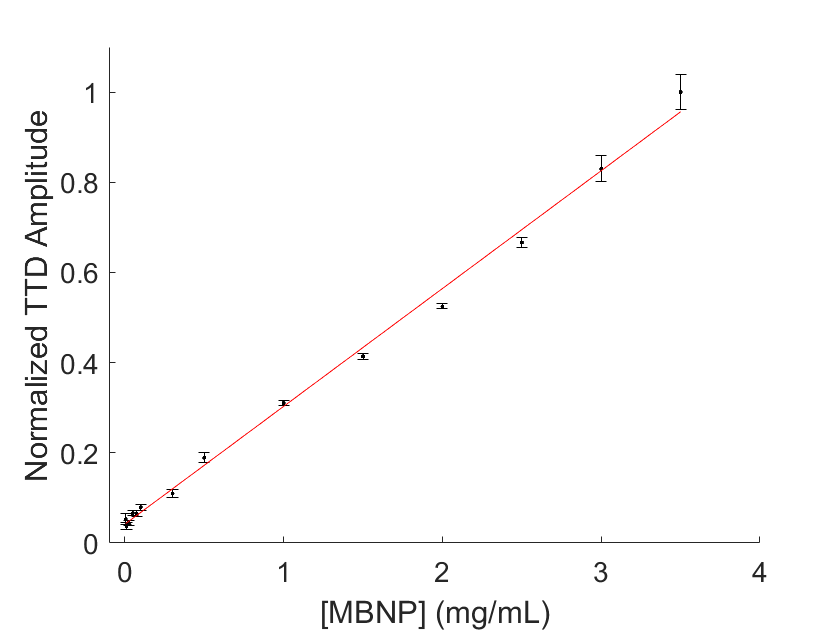


**Figure S3.** TTD signal vs concentration of MBNP in the presence of 10% blood. The linear concentration dependence of the TTD signal is unaffected by the presence of other chromophores such as Hb and HbO_2_.

**
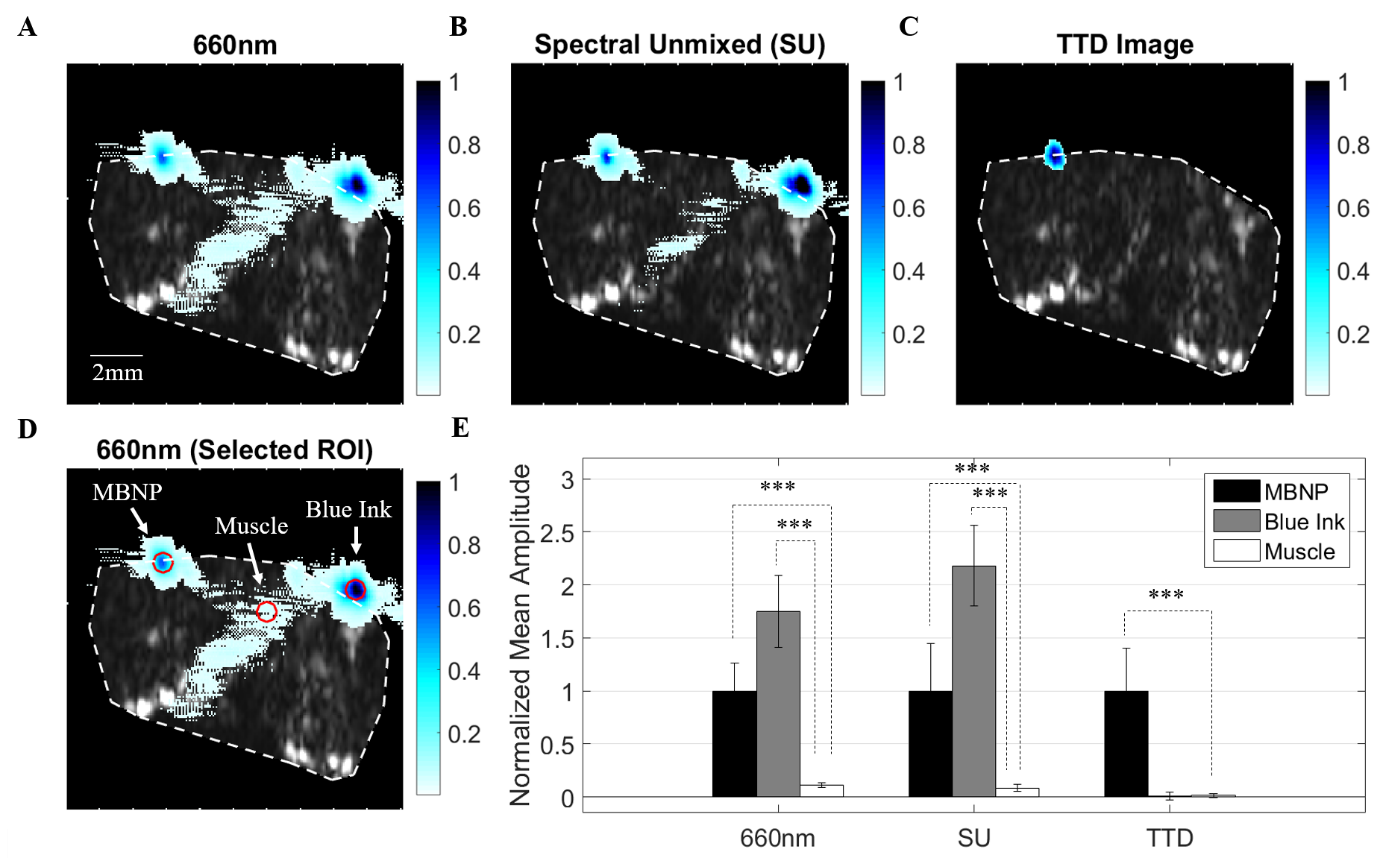
**

**Figure S4.** PA imaging results on a rat muscle where a drop of MBNP and a drop of blue ink were added to the top surface of the muscle tissue (MBNP at the left; blue ink at the right). Here, the PA images superimposed on a gray-scale ultrasound image are shown for **(A)** the raw MBNP signal taken at its absorption peak of 660 nm, **(B)** the spectral unmixed signal detailing the estimated concentration of MBNP, and **(C)** the TTD image. The white dotted line details the muscle tissue outline. **(D)** shows the region-of-interest (ROI) selected for quantitative analysis (outlined in red), centered at the maximum intensity with size chosen to match expected sample drop size. Colorbar shows the normalized intensity based on the maximum intensity within each imaging method. **(E)** shows the normalized signal amplitude for the selected ROI for the three different methods. Error bars indicate the standard deviation within each selected ROI. *** represents p<0.001 for a t-test with hypothesis that the normalized signal amplitude for the selected ROI is equal to that of the muscle (control) ROI.
